# Recent enhanced high-summer North Atlantic Jet variability emerges from three-century context

**DOI:** 10.1038/s41467-017-02699-3

**Published:** 2018-01-12

**Authors:** V. Trouet, F. Babst, M. Meko

**Affiliations:** 1 Laboratory of Tree-Ring Research, University of Arizona, 1215 East Lowell Street, Tucson, AZ 85721 USA; 2 Landscape Dynamics Unit, Swiss Federal Research Institute WSL, Zürcherstrasse 111, Birmensdorf, CH-8903 Switzerland; 3 W. Szafer Institute of Botany, Polish Academy of Sciences, ul. Lubicz 46, Krakow, 31-512 Poland

## Abstract

A recent increase in mid-latitude extreme weather events has been linked to Northern Hemisphere polar jet stream anomalies. To put recent trends in a historical perspective, long-term records of jet stream variability are needed. Here we combine two tree-ring records from the British Isles and the northeastern Mediterranean to reconstruct variability in the latitudinal position of the high-summer North Atlantic Jet (NAJ) back to 1725 CE. We find that northward NAJ anomalies have resulted in heatwaves and droughts in northwestern Europe and southward anomalies have promoted wildfires in southeastern Europe. We further find an unprecedented increase in NAJ variance since the 1960s, which co-occurs with enhanced late twentieth century variance in the Central and North Pacific Basin. Our results suggest increased late twentieth century interannual meridional jet stream variability and support more sinuous jet stream patterns and quasi-resonant amplification as potential dynamic pathways for Arctic warming to influence mid-latitude weather.

## Abstract (web summary)

Long-term records of jet stream variability are needed to place recent mid-latitude extreme weather events into a historical context. Here, using tree-ring records from Europe, the authors reconstruct variability in the latitudinal position of the high-summer North Atlantic Jet since 1725 CE.

## Introduction

The position and strength of the Northern Hemisphere polar jet stream are important modulators of mid-latitude weather extremes^1^ and their societal, ecosystem, and economic impacts^2^. In Europe, the position of the North Atlantic Jet (NAJ) drives temperature and precipitation extremes^3,4^ by controlling the location of the Atlantic storm track and by influencing the occurrence and duration of near-stationary atmospheric pressure fields (“atmospheric blocking”). The NAJ is strongest in winter^5^, but also influences European climate in summer, the season that is most commonly recorded in tree rings as climate proxies. In summer, a southern NAJ regime is associated with a decrease in blocking frequency over the British Isles (BRIT) and northwestern Europe that can result in floods in these regions^6^. In the northeastern Mediterranean (NEMED), on the other hand, the southern NAJ regime phase increases blocking frequency as well as the odds of heatwaves^3^. Such conditions occurred during the summer of 2007, when BRIT experienced the second wettest summer on record since 1912^7^, whereas record-breaking high temperatures—with departures from the seasonal means exceeding 4 °C in some areas^8^—were observed across NEMED that led to excess human mortality and prompted catastrophic wildfires^9^. In contrast, the summer of 1976—when the NAJ was in an anomalously northward position and blocking patterns were reversed—was one of the driest and hottest summers on record in BRIT, but was associated with a NEMED cold extreme^10^. General circulation models (GCMs) systematically overestimate the seasonal cycle in the NAJ latitudinal position about the mean, with the majority of simulations showing a poleward bias in summer NAJ position compared to observational records^11,12^. GCM simulations largely agree, however, in their projection of the mean position of the mid-latitude jet stream to shift poleward in future climates with increased anthropogenic forcing^11^ and this result is particularly robust for the NAJ and for the summer season^13,14^.

An exceptional number of mid-latitude extreme weather events over the last decade has encouraged a suite of observational and modeling studies investigating the relative role of anthropogenic climate forcing and natural variability in driving this recent increase in weather extremes^2,4,15^. One hypothesis suggests variability in the amplitude and speed of the Northern Hemisphere jet stream as a potential mechanism linking recent mid-latitude weather extremes to anthropogenic warming^16–18^. This hypothesis is largely based on statistical associations between observational or atmospheric reanalysis data that are supported by plausible physical mechanisms. However, the data sets most frequently used provide relatively short time series (1979–present for the satellite era, 1948–present for the reanalysis era) that do not warrant robust results from a statistical significance perspective and that hamper the detection of non-linear relationships in a complex climate system^19^. Long-term records of jet stream variability are thus needed to put recent trends in a historical perspective and to investigate non-linear relationships between jet stream variability, mid-latitude extreme weather events, and anthropogenic climate change^17,18^.

Here we reconstruct interannual variability in the latitudinal position of the August NAJ back to 1725 CE by combining two summer temperature-sensitive tree-ring records. We find that extreme weather events—including floods, heatwaves, and wildfires—in BRIT and NEMED over the past 300 years have been linked to August NAJ anomalies. Our NAJ reconstruction shows that late twentieth century NAJ positions fall within the range of the preceding centuries, but that a recent increase in the number of NAJ anomalies is unprecedented. This increase in NAJ variance coincides with enhanced variance in the Pacific Basin and points to an increase in interannual meridional jet stream variability since the 1960s.

## Results

### Tree-ring-based reconstruction of August NAJ variability

**Fig. 1:**
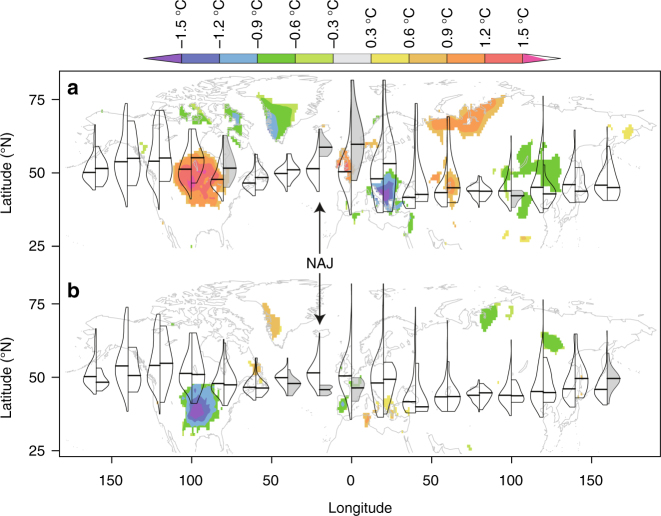
Latitudinal position of the August Northern Hemisphere Jet. Left wings of the violins represent the August Northern Hemisphere Jet latitudinal position distribution over the instrumental period (1920–2012) for 20° longitudinal slices. Right wings represent distribution during anomalous years when the North Atlantic Jet (NAJ; 10–30°W) latitudinal position exceeded 1 stdev northwards (**a**) or southwards (**b**). Gray shading indicates significant differences between the left and right distributions (one-sided Kolmogorov–Smirnov test; *p* < 0.05). Background map shows August surface temperature anomalies (°C; CRUTEM3.21^60^) composited over the anomalous years. Composite maps were created in R with color palette adapted from the KNMI Climate Explorer (https://climexp.knmi.nl)

Variability in the position of the summer NAJ is linked to jet stream variability over Central North America, Europe, and eastern Asia^3^ (Fig. 1). Jet anomalies in the eastern North Atlantic (10–30W)—the region that comprises the Icelandic Low and Azores High centers of action—are most influential for European climate (Fig. 1, Supplementary Figs. 1-4). Northern (southern) jet anomalies in this region correspond to northern (southern) jet anomalies over northwestern Europe and southeastern Europe and create a summer temperature seesaw between BRIT and NEMED (Fig. 1; Supplementary Figs. 1, 4b). August jet anomalies in the eastern North Atlantic are further representative for a broader temporal window (July and August) and a broader geographical range in the North Atlantic (58W to 8E; Supplementary Fig. 2 in ref.^20^).

**Fig. 2:**
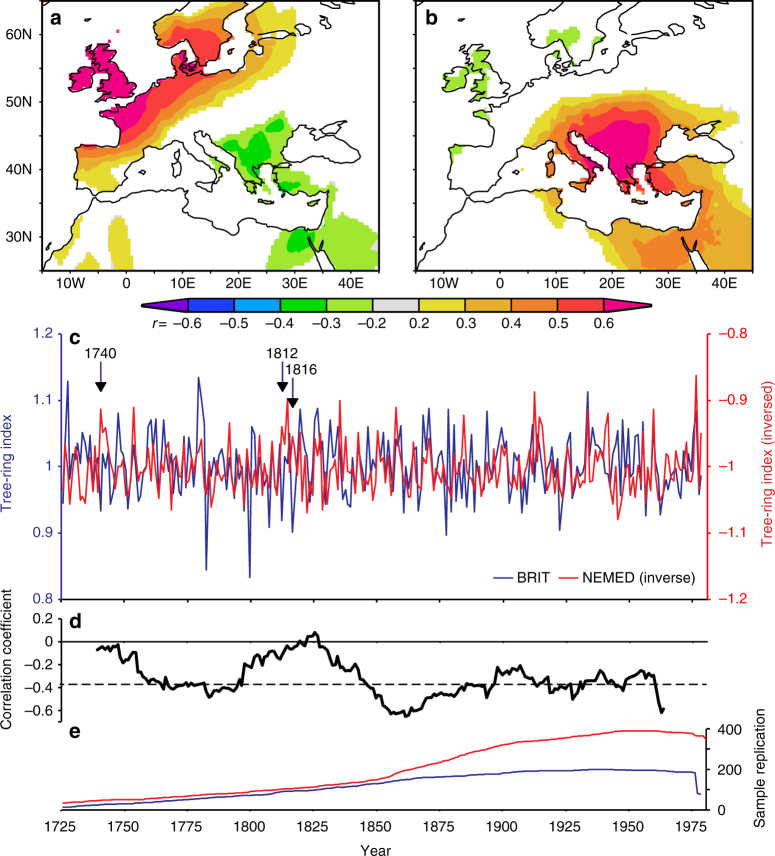
BRIT and NEMED tree-ring chronologies. **a**, **b** Pearson's correlation maps of BRIT (**a**) and NEMED (**b**) tree-ring chronologies with gridded 1° CRU TS4.0^47^ August temperature anomaly fields (1901–1978) over Europe. Correlation coefficients higher than 0.3 are significant at the *p* < 0.01 significance level. **c** BRIT and NEMED (inversed) tree-ring chronologies (1725–1978) and **d** their sample replication over time. **e** The 31-year running Pearson's correlation coefficients between the BRIT and NEMED chronologies are consistently negative over the full period, except for years (1740, 1812, 1816) when external forcings created cold conditions throughout Europe. Correlation coefficients below −0.374 (dashed line) are significant at the *p* < 0.05 level. Correlation maps in **a**, **b** were created in the KNMI Climate Explorer (https://climexp.knmi.nl)

The temperature seesaw created by August NAJ anomalies is reflected in regional tree-ring records^10^ (Fig. 2a, b, Supplementary Figs. 4c, 5b). We have compiled maximum latewood density (MXD) records for both regions (Supplementary Table 1) that represent interannual variability in regional August average surface temperature (Fig. 2a, b; Supplementary Fig. 5a). The two well-replicated (Fig. 2d) temperature proxies explain 52% (*r* = 0.72, *p* < 0.001; 1901–1978; Fig. 2a) and 44% (*r* = 0.66, *p* < 0.001; 1901–1980; Fig. 2b) of the variance in regional August average temperature variation in BRIT and NEMED, respectively, and thus illustrate the temperature dipole generated by anomalous NAJ positions. The BRIT and NEMED tree-ring records correlate significantly negatively with each other over their period of overlap (*r* = −0.29; *p* < 0.01; 1725–1978; Fig. 2c) and the negative correlation between the two tree-ring series weakens only in years when volcanic (e.g., Mount Tabora in 1816; *r* = −0.06) or other forcings (e.g., in 1740, *r* = −0.05) generate cold summers over the entire European continent (Fig. 2e).

**Fig. 3:**
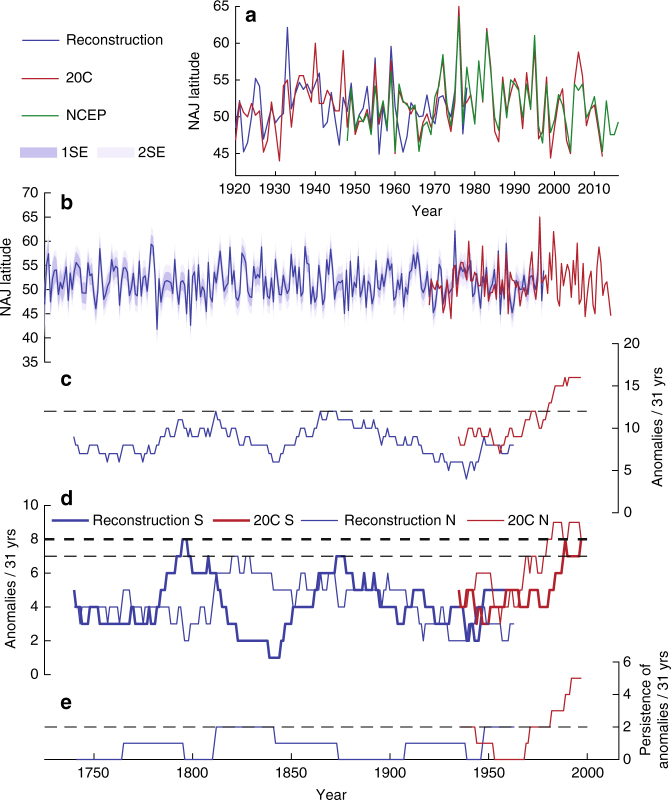
Summer NAJ reconstruction and variance. The reconstruction of the latitudinal position of the August NAJ was scaled and calibrated against NAJ position calculated based on twentieth century reanalysis data and explains 40% of its variance over the period of overlap (1920–1978; **a**). The NCEP/NCAR reanalysis data (1948–2016) are plotted for comparison in **a**. The full NAJ reconstruction (1725–1978) including combined error estimations is plotted in **b**. Running 31-year window number of NAJ anomalies (**c**), number of northern (N) and southern (S) NAJ anomalies (**d**), and persistence of anomalies (**e**) are plotted on the central year of the window for reconstructed (blue) and 20C Reanalysis (red) NAJ time series. Anomalies are defined as years when NAJ >1 stdev, with standard deviation calculated based on a merged time series of reconstructed (1725–1919) and 20C Reanalysis (1920–1978) NAJ values. Horizontal dashed lines in **c**–**e** represent the highest 31-year values over the reconstruction period (1725–1978)

The BRIT and NEMED MXD chronologies are both significantly correlated with August NAJ position (*r* = 0.5 and *r* = −0.57, respectively; *p* < 0.001; 1920–1978) and their composite explains close to 40% of the variance in the August NAJ target (*r* = 0.63;* R*^2^_a_ = 0.39; *p* < 0.01; Fig. 3a). Calibration and verification trials show that August NAJ position can be skillfully reconstructed back to 1725 based on this combination (Fig. 3b), with 27 to 52% of the variance explained in the verification procedure and overall positive reduction of error (RE) and coefficient of efficiency (CE) statistics (Supplementary Table 2). Calibration/verification statistics, however, were weaker for the early calibration period (1920–1948) compared to the later period (1949–1978), which is possibly due to inhomogeneities in the earliest period of the twentieth century reanalysis target data set^21^.

### 300 years of high-summer NAJ variability

The reconstruction shows that late twentieth century NAJ positions fall within the latitudinal NAJ range of the preceding centuries, with the exception of the summer of 1976, which was the northernmost NAJ position in both the instrumental data and the reconstruction. The southernmost NAJ position of the target time series (44N in 1931) was exceeded only twice in the late eighteenth century (1782 and 1799; Fig. 3b). These two years respectively are the seventh and second wettest Augusts in England and Wales since record keeping began in 1766^22^. It is worth noting that the NAJ can deviate further northward from its average position (51.6N) than southward (Fig. 1, Supplementary Fig. 1) and as a result European climate anomalies can be more extreme during northern compared to southern NAJ anomalies (Fig. 1, Supplementary Figs. 1-3).

NAJ variability is often characterized using circulation indices such as the North Atlantic Oscillation (NAO) and the East Atlantic (EA) pattern, which describe combined changes in NAJ position and speed^14,20,23^. When the NAJ is in an anomalously northerly position, it generates stronger than normal cyclonic conditions to the north (Icelandic Low) and anticyclonic conditions to the south (Azores High), corresponding to positive NAO and negative EA phases. Our August NAJ target correlates significantly with summer NAO (*r* = 0.37, *p* < 0.01) and EA (*r* = −0.49, *p* < 0.01) indices. The NAJ reconstruction also correlates significantly positively with a tree-ring-based summer NAO reconstruction^24^ over their full length of overlap (1725–1976; *r* = 0.59, *p* < 0.01; Supplementary Fig. 6). The two reconstructions are not fully independent, with 13% of the tree-ring series used in the NAJ reconstruction contributing to the summer NAO reconstruction, but still demonstrate the long-term linkages between North Atlantic summer atmospheric circulation and jet stream variability.

**Fig. 4:**
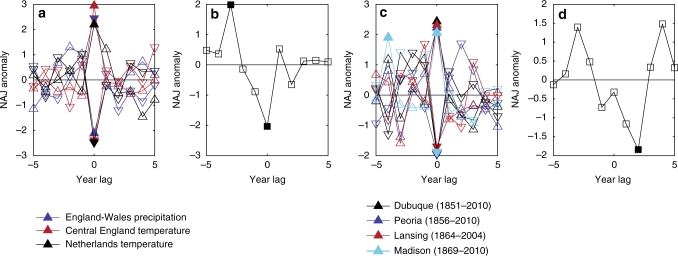
NAJ positions during historical weather extremes and volcanic eruptions. Superposed epoch analyses (SEA) of NW Europe climate (**a**), SE Europe fire (**b**), North American temperature (**c**), and volcanic eruptions (**d**) event series with August NAJ reconstruction. Event series used are the England-Wales summer (June–August) precipitation (**a**; 1766–2014) and Central England summer temperature (**a**; 1725–2014) time series^22^, the Netherlands summer temperature time series^58^ (**a**; 1725–2000), a tree-ring-based fire record from Mt. Taygetos, Greece^29^ (**b**; 1823–1940), August temperature data from four meteorological stations in North America (Supplementary Fig. 1) with records dating back to the nineteenth century (**c**), and an ice-core-based volcanic event series^59^ (**d**; *n* = 25, 1725–1900). Filled symbols indicate statistical significance (*p* < 0.05). Event years in **a** and **c** are defined as >1.5 stdev (upward triangle) and <1.5 stdev (downward triangle) of the average of the time series. The analysis window includes up to 5 years before and after each event year

We used existing MXD data for our NAJ reconstruction, primarily collected by other researchers (Supplementary Table 1), which allowed us to optimize sample replication and climate sensitivity, but limited our ability to retain low-frequency variability (see Methods section). As a result, BRIT, NEMED, and the NAJ reconstruction are dominated by sub-decadal variability (~4 to 6 years; Supplementary Fig. 7) and no significant long-term poleward or equatorward trends were detected (Fig. 3b). We found no significant relationship between reconstructed NAJ position and past El Niño Southern Oscillation (ENSO) events, but found a southward NAJ shift 2 years after past volcanic eruptions (Fig. 4d). Furthermore, the NAJ time series shows a steep and unprecedented increase in the number of NAJ anomalies—and thus in variance—starting in the 1960s (Fig. 3c). An analysis of northward (positive) and southward (negative) anomalies separately (Fig. 3d) shows that anomalies of both signs are more frequent since the 1960s, but only the number of northward anomalies reaches unprecedented levels starting ca. 1980. The late twentieth century synchronicity by itself is unprecedented, however, with previous centuries showing a seesaw in the number of northward versus southward anomalies (Fig. 3d). Such a simultaneous increase of northward and southward anomalies and thus increased interannual meridional variability is likely indicative of a stronger NAJ “wobbling”^13^ and a more sinuous NAJ, rather than a southward (~1785–1810 CE) or northward (~1810–1870 CE) shift of the NAJ regime, as might have happened in the past (Fig. 3d). The increased number of anomalies further results in an enhanced persistence of anomalies, defined as the number of occasions when anomalies of the same sign occurred in consecutive years (Fig. 3e). Persistence of weather extremes related to such NAJ anomalies can be particularly challenging for agricultural and hydropower productivity and for natural ecosystems.

Our NAJ reconstruction provides a multi-century benchmark against which to test the late twentieth century increase in occurrence and persistence of anomalies that is visible in the reanalysis NAJ time series (Fig. 3c–e). The unprecedented character of the late twentieth century increase is independent of reconstruction scaling period (Supplementary Fig. 8), but we recognize that our NAJ reconstruction ends in 1978 and thus does not itself register the increase. Nevertheless, the NAJ reconstruction is scaled to the variance of the instrumental target and reconstruction and target are characterized by very similar skewness (0.9 and 0.94, respectively) and kurtosis (4.78 and 4.58, respectively) values over their period of overlap (1920–1978), suggesting that the NAJ variance is well represented by the reconstruction (Supplementary Fig. 9). More recent tree-ring collections are available for BRIT^25^ and NEMED^26^ that in future analyses can be used to measure MXD, then reconstruct regional summer temperature, extend the NAJ into the twenty-first century, and capture late twentieth century NAJ variance. High mean segment lengths (e.g., 400+ years for NEMED) and quasi-millennial-length time series (800+ years for BRIT, 1000+ years for NEMED) will allow to retain more low-frequency variability, to investigate decadal-scale drivers of summer NAJ variability such as the Atlantic Multidecadal Oscillation and sea ice fluctuations^23^, and to study centennial-scale NAJ variability during the Medieval Climate Anomaly and Little Ice Age^27^.

Extreme summer weather events in BRIT, NEMED, and the American Midwest are often associated with northward and southward NAJ deviations^3,4,6^. A comparison of our NAJ reconstruction with independent time series of past BRIT summer weather and NEMED fire occurrence (Fig. 4a, b) confirms that the position of the NAJ has impacted European extreme weather events since at least 1725 CE. Heatwaves and droughts in BRIT and the northwestern European Lowlands have consistently been associated with northern NAJ positions, whereas the NAJ showed southward anomalies during pluvials and cold summers (Fig. 4a). For instance, the southernmost NAJ position in the reconstruction occurred in 1782 (Fig. 3b), which was such a cold summer in Scotland that the grain harvest failed and famine arose^28^. A long rainy spell dominated the second most southern summer of 1799 in England, with only 8 days without rain between 22 June and 17 November^28^. August temperature data from four meteorological stations with records dating back to the nineteenth century (Supplementary Fig. 1) indicate that in the American Midwest, heatwaves have also consistently occurred during northern and summer cold spells during southern NAJ positions (Fig. 4c).

In NEMED, nineteenth and early twentieth century fire events occurred during southern NAJ summers (Fig. 4b) and hence during wet, cold BRIT summers (Supplementary Fig. 10a). Late summer (July–August) is the dominant fire season in NEMED forests^29^ and a southern NAJ position in this period creates hot and dry conditions that are favorable for wildfire occurrence and spread. Moreover, northern NAJ positions 3-year prior also induce fire conditions (Fig. 4d, Supplementary Fig. 10b), which could be due to the quasi-periodic character of NAJ with a 6-year peak (Supplementary Fig. 7b). However, such lagged moisture/fire relationships are also prominent in fire regimes in the American Southwest^30^, where preceding cool and wet summers increase fuel load and promote burning in subsequent dry summers, and could thus be prevailing in NEMED as well. Increased NAJ variance—with both northern and southern NAJ anomalies occurring more frequently—can thus create NEMED climate conditions that are conducive to widespread burning. Unlike in the American Southwest, however, the historical NEMED fire record is extremely sparse^29^ and additional regional paleofire records are needed to fully characterize NEMED fire–climate relationships.

## Discussion

**Fig. 5:**
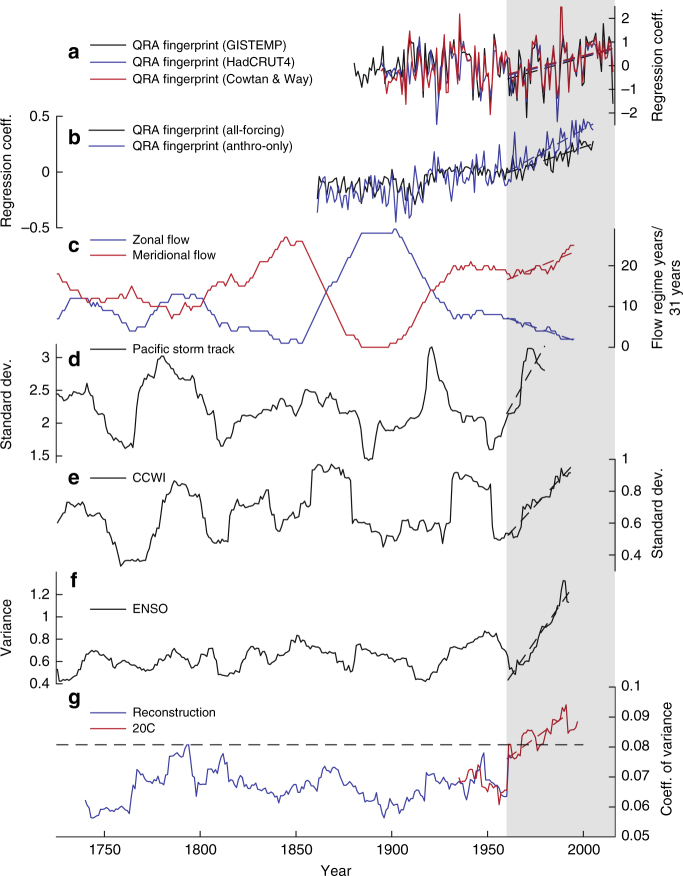
Late twentieth century variance increase in North Atlantic and North Pacific Basin. Running (31-year window) coefficients of variance of August NAJ time series (**g**) are compared to time series of observed (**a**) and modeled (**b**) quasi-resonant amplification (QRA) fingerprint^15^, of reconstructed zonal and meridional flow in the North Pacific^36^ (**c**), and of variance in the climate dynamics of the North (**d**,** e**) and Central (**f**) Pacific. Variance time series include the Pacific storm track^33^ (31-year standard deviation; **d**), California Current Winter Index^32^ (CCWI; 31-year standard deviation; **e**), and NIÑO4 SST index^31^ (31-year variance; **f**). The late twentieth century increased variance period (1960–present) is highlighted in gray. Horizontal dashed line in **g** represents the highest 31-year values over the reconstruction period (1725–1978 CE)

Enhanced late twentieth century variance has also been detected in climate and ecosystem dynamics in the Central^31^ and Northeast Pacific^32,33^ (Fig. 5d–f). Climate dynamics in the Central and Northeast Pacific are strongly linked to the ENSO and the Pacific North American patterns, two drivers of atmospheric circulation variability that in turn are associated with the latitudinal position of the North Pacific Jet (NPJ)^20,34,35^. Our combined results (Fig. 5) thus suggest a late twentieth century increase in jet stream latitudinal variance in the North Atlantic and the North Pacific Basin, the two basins where interannual jet stream variability is strongest^20^. CMIP5 projections indicate that such meridional variability will weaken on a daily time scale as mid-latitude jets move to higher latitudes with increasing greenhouse gas emissions^13^. However, on interannual time scales, increased meridional jet stream variability can reflect a wavier, slower jet that is accompanied with blocking systems and thus creates contrasting mid-latitude extreme weather events^23^. The enhanced NAJ variability further coincides with a prevalence of meridional flow regimes and an infrequency of zonal flow in the North Pacific Basin^36^ (Fig. 5c). The larger north–south NAJ and NPJ deviations and the more meridional flow can be indicative of enhanced jet stream waviness^16^ and coincide with a modeled and observed increase in recent decades in conditions that are favorable to the occurrence of quasi-resonant amplification^15^ (QRA; Fig. 5a, b). QRA is the process through which synoptic-scale Rossby waves (with zonal wave numbers 6–8) are trapped within an effective mid-latitude atmospheric waveguide and create persistent extreme mid-latitude summer weather in the Northern Hemisphere^37^. QRA as well as a more sinuous jet stream can result from a decrease in the equator-to-pole temperature gradient and thus from Arctic amplification—the disproportionate warming of the Arctic region compared to the mid-latitudes over the past three decades. Both mechanisms have been proposed as potential dynamical pathways linking recent extreme mid-latitude weather events to Arctic warming^1,15,17,18,37^. This, however, is a topic of intense scientific debate^18,19^ because the link between Arctic amplification and increased mid-latitude blocking pattern frequencies is sensitive to the data set used, the choice of geographical region, and the metrics describing wave activity and blocking patterns^17,19,38^. Our results contribute to this debate and support more sinuous jet stream patterns and QRA as potential dynamic pathways for Arctic warming to influence mid-latitude weather. North Atlantic and North Pacific tree-ring-based reconstructions of jet stream-related indices show a synchronous late twentieth century amplification of the meridional component^36^ and of variance (Fig. 5) that can generate more frequent mid-latitude blocking patterns and facilitate persistent periods of extreme weather, including wildfires. Moreover, the synchronization of variance increases between the North Atlantic and North Pacific basins in the late twentieth century is unprecedented over the last 290 years (Fig. 5) and strongly suggests an impact of anthropogenic warming.

## Methods

### Climate data

We calculated the latitudinal position of the Northern Hemisphere polar jet (NHJ) based on 300 hPa scalar wind data (m s^−1^) averaged for August and derived from the twentieth century reanalysis data set^39^. This data set contains globally gridded (2°x2°) monthly pressure values for the period 1871–2010, but is likely affected in its earliest period by inhomogeneity due to decline in station density and measurement quality^5,21^. We therefore restricted our time series derived from this data set to the period 1920–2012, which includes an adequate time frame (1920–1978) for statistically significant calibration/verification exercises^40^. August NHJ position for each longitudinal grid cell was defined as the latitude with the greatest average August 300 hPa scalar wind speed^41,42^ and NAJ positions were averaged over a subset of longitudes (10–30W) in the eastern North Atlantic.

For the period 2013–2016, we calculated August NAJ position based on the NCEP/NCAR Reanalysis data set^43^, which includes globally gridded (2.5°x2.5°) monthly pressure values for the period 1948–present. NAJ position was calculated in the same way as described above for the twentieth century reanalysis data set and the two data sets correlate strongly (*r* = 0.93, *p* < 0.001) over their period of overlap (1948–2012 CE). The NCEP/NCAR NAJ data were then linearly transformed (also referred to as scaled) to match the mean and standard deviation of the twentieth century reanalysis NAJ data. Mean and standard deviation for scaling were calculated over the period that was common to the two instrumental data sets and the tree-ring data set (1948–1978 CE). We further calculated Pearson's correlation coefficients between August NAJ position and rotated principal component analysis-based indices of summer (June–August) NAO and EA^44^ as provided by the NOAA Climate Prediction Center (1950–2012 CE).

### Tree-ring data

MXD tree-ring data for BRIT and NEMED were selected from the International Tree Ring Data Bank^45^ (http://www.ncdc.noaa.gov/paleo/paleo.html). The NEMED compilation^46^ (1675–1980) contains 401 individual MXD series from four tree species at 21 high-elevation sites (Supplementary Table 1) and explains 44% of the variance in regional August average temperature variation (*r* = 0.66, *p* < 0.001; 1901–1980; Fig. 2b, Supplementary Fig. 5a), calculated based on a regional average (38–45N, 15–25E) of a monthly gridded August temperature field^47^ (1°; CRU TS4.0). For BRIT, we compiled 213 individual Scots pine (*Pinus sylvestris*) MXD series from 11 sites (Supplementary Table 1) to develop one BRIT master chronology (average inter-series correlation = 0.531, mean segment length = 147 years) that explains 52% of the variance in regional (47–57N, 10W–2E) August temperature variation (*r* = 0.72, *p* < 0.001; 1901–1978; Fig. 2a; Supplementary Fig. 5a).

Age-related trends were removed from all raw measurements using cubic smoothing splines with a 50% frequency-response cutoff equal to 100 years^48^ and two regional chronologies (BRIT and NEMED) were then calculated by averaging the detrended series using a biweight robust mean^49^. The chronologies had estimated population signal values^50^ above the commonly utilized, but arbitrary, threshold of 0.85 from 1675 (NEMED) and 1725 (BRIT) onwards.

### Reconstruction

We applied a composite-plus-scale method^51^ to reconstruct August NAJ position from our BRIT and NEMED MXD chronologies. We first normalized the two chronologies using the mean and standard deviation over their full period of overlap (1725–1978 CE) and then calculated the average of BRIT and negative NEMED for each year. The resulting BRIT–NEMED composite is strongly and significantly positively correlated with August and August–September average NAJ position over longitudes between 10 and 30W (Supplementary Fig. 5b). Because the longitudinal window of significant correlations is wider for August NAJ than for the August–September average (Supplementary Fig. 4b) and because the BRIT MXD chronology is only weakly correlated with regional September temperatures (Supplementary Fig. 5a), we chose August NAJ position for the longitudes between 10 and 30W as our target for reconstruction. We reconstructed August NAJ position by scaling the BRIT–NEMED composite to match the mean and variance of the August NAJ data over the period 1948–1978 CE^52^. Calibration/verification tests—including RE and CE statistics^53^—were calculated on the scaled reconstruction over two subperiods (1920–1949 and 1950–1978 CE; Supplementary Table 2). Uncertainty in the NAJ reconstruction arises from unexplained variance in the scaling model (calibration error) and the decreasing number of tree-ring series back through time^54^ (chronology error). We estimated the calibration error of the scaling model as two standard error 95% confidence intervals (CIs) for the full calibration period 1920–1978 CE. The chronology error was estimated by bootstrapping^55^: standardized tree-ring measurements were sampled with replacement 1000 times and arithmetic means were calculated. Two-tailed 95% CIs were estimated based on the distribution of the bootstrapped mean and scaling technique was then applied to the upper and lower CI limits. The overall error for the NAJ reconstruction was estimated as the square root of the summed and squared calibration and chronology error terms.

### Variance and extreme analysis

We used three metrics to calculate variability in NAJ variance over time. First, we calculated 31-year window running coefficients of variance (standard deviation (stdev)/average) for the NAJ reconstruction and for the 20C reanalysis data (Fig. 5g). We also calculated for both time series the running number of extreme NAJ anomalies per 31-year period (Fig. 3c) and the number of occasions per 31-year period when NAJ extremes of the same sign occurred in consecutive years (persistence of extremes; Fig. 3e). Extreme anomalies for both time series were defined as years when NAJ exceeds 1 stdev (positive or negative), with stdev calculated based on a merged time series of reconstructed (1725–1919) and 20C Reanalysis (1920–1978) NAJ values. The running number of extreme NAJ anomalies per 31-year period was also calculated for positive and negative extremes separately (Fig. 3d).

Superposed epoch analysis (SEA) was used to evaluate the relationship between NAJ position and extreme weather events in BRIT, NEMED, and the American Midwest, as well as past volcanic eruptions and ENSO events (results not shown) by superposing a window of contemporaneous and lagged NAJ positions over each extreme weather or volcanic event^56^ (Fig. 4). Significance levels were determined from bootstrapped 95% CI estimates based on Monte Carlo simulations^57^. Extreme weather events in BRIT (Fig. 4a) and the American Midwest (Fig. 4c) and ENSO events were defined as values >1.5 stdev and <1.5 stdev of the average of the time series. For BRIT we used time series of Central England summer (June–August) temperature (1725–2014), England-Wales summer precipitation^22^ (1766–2014), and Netherlands summer temperature^58^ (1725–2000). For the American Midwest we extracted August temperature time series from four meteorological stations with records dating back to the nineteenth century (Supplementary Fig. 1). ENSO events were determined based on the Liu et al.^31^ time series. NEMED extreme events were fire years derived from a tree-ring-based fire record from Mt. Taygetos in Greece^29^. Fire years (*n* = 12; 1823–1940 CE) were defined as the years prior to 1940 when two or more recording trees were scarred. We did not use the most recent period (1940–2010) of the record^29^ because Second World War (1940–1945) battles in the mountain range and the subsequent establishment of the Forest Service as well as land-use changes^9^ have left a strong human fingerprint on recent wildfires in the region. Past volcanic eruptions (*n* = 25, 1725–1900 CE) were determined based on the Toohey and Sigl^59^ time series.

### Data availability

The August NAJ reconstruction will be housed upon publication with NOAA-Paleoclimatology/World Data Service for Paleoclimatology: https://www.ncdc.noaa.gov/data-access/paleoclimatology-data. The BRIT and NEMED MXD chronologies are available from the corresponding author on reasonable request.

## Electronic supplementary material

Supplementary Information
