# Supplementary material for: Recent enhanced high-summer North Atlantic Jet variability emerges from three-century context
Source: Nat Commun. 2018 Jan 12;9:180. doi: 10.1038/s41467-017-02699-3 (PMC5766518; doi:10.1038/s41467-017-02699-3)
Supplement: Supplementary file 1 — Supplementary Information [file 41467_2017_2699_MOESM1_ESM.pdf]

**Supplementary Figure 1: Latitudinal position of the August Northern Hemisphere Jet (NHJ).** *(same figure as Fig. 1 but for 1.5 stdev anomalies in stead of 1 stdev)* Left wings of the violins represent the August NHJ latitudinal position distribution over the instrumental period (1920-2012) for 20° longitudinal slices. Right wings represent distribution during anomalous years when the North Atlantic Jet (NAJ; 10-30° W) latitudinal position exceeded 1.5 standard deviations northwards (a) or southwards (b). Gray shading indicates significant differences between the left and right distributions (one-sided Kolmogorov-Smirnov test; $p<0.05$). Background map shows August surface temperature anomalies (°C; CRUTEM3.21[1]) composited over the anomalous years. Black dots indicate the location of meteorological stations used in Fig. 4c. Composite maps created in R with color palette adapted from the KNMI Climate Explorer (https://climexp.knmi.nl).

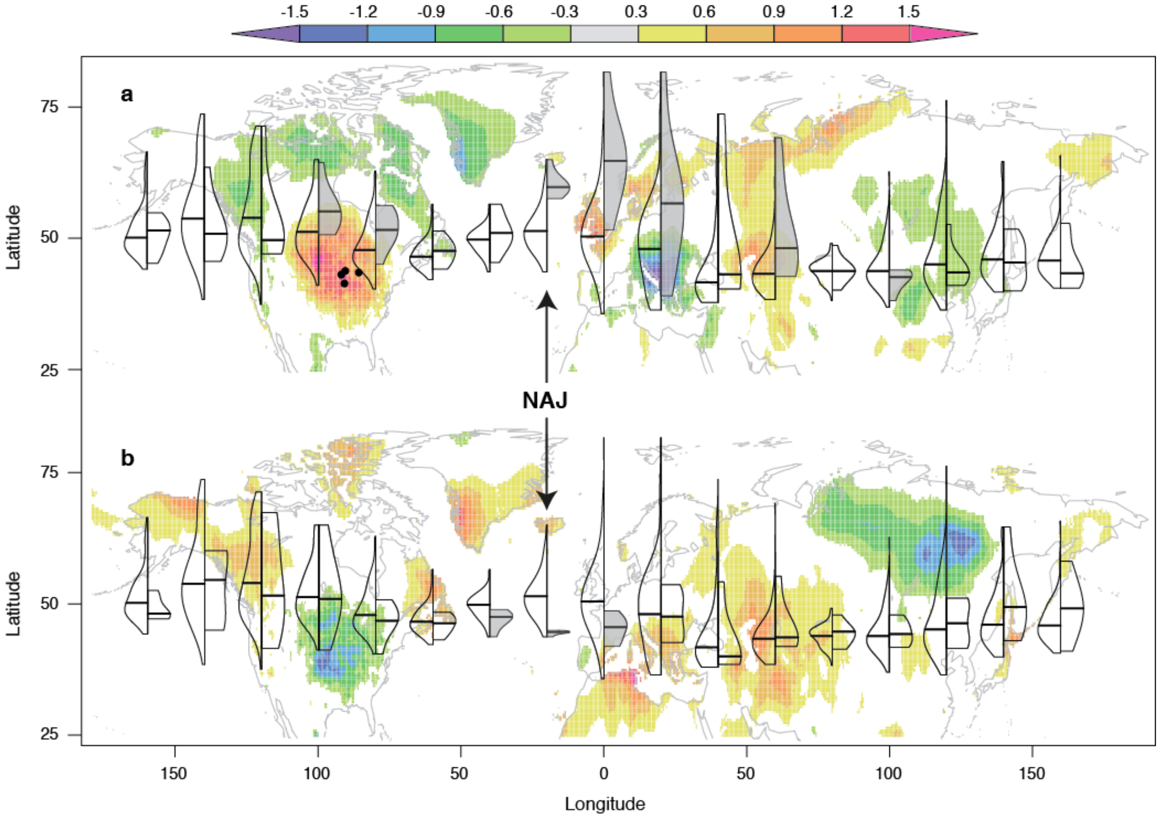

-1.5
-1.2
-0.9
-0.6
-0.3
0.3
0.6
0.9
1.2
1.5
a
b
NAJ
Latitude
75
50
25
Latitude
75
50
25
150
100
50
0
50
100
150
Longitude

**Supplementary Figure 2:** August precipitation anomalies (mm; CRUTEM3.21[1]; 1920-2012] composited over anomalous years when the North Atlantic Jet (NAJ; 10-30° W) latitudinal position exceeded 1.5 standard deviations northwards (a) or southwards (b). Non-significant results have been masked out. Composite maps created in R with color palette adapted from the KNMI Climate Explorer (https://climexp.knmi.nl).

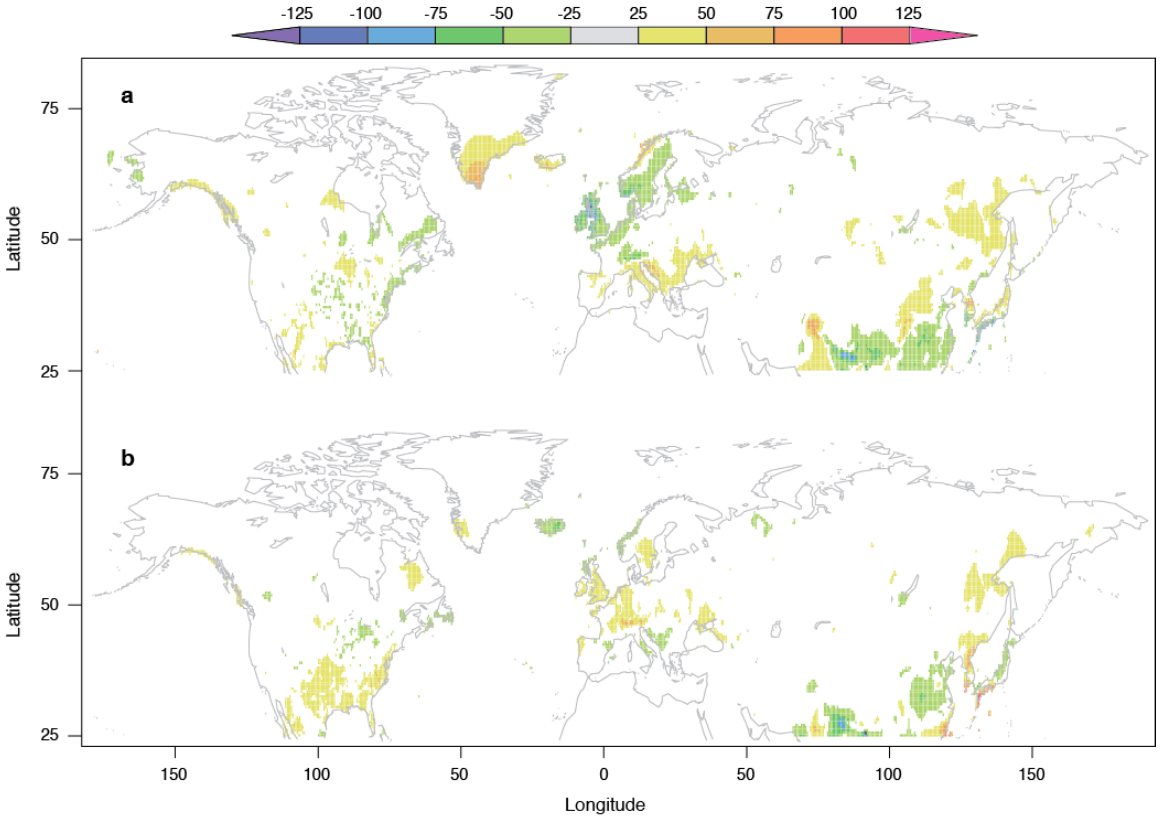

**Supplementary Figure 3:** August 500 hPa geopotential height anomalies (Twentieth Century Reanalysis[2]; 1920-2012] composited over anomalous years when the North Atlantic Jet (NAJ; 10-30° W) latitudinal position exceeded 1 standard deviations southwards (a) or northwards (b). Non-significant (P>0.05) results have been masked out. Composite maps created in the KNMI Climate Explorer (https://climexp.knmi.nl).

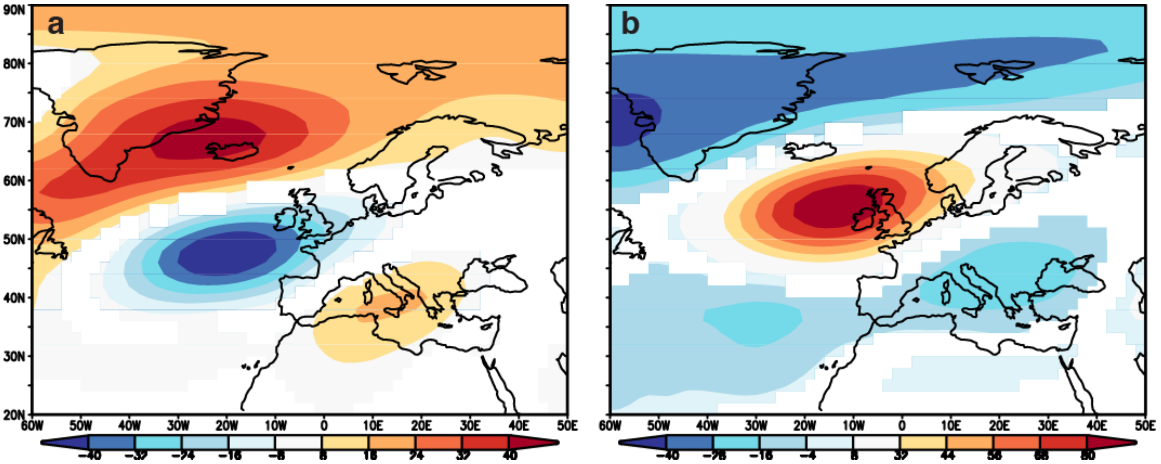

**Supplementary Figure 4:** Correlation coefficients of NEMED (38-45N; 15-25E) and BRIT (47-57N; 10W-2E) (a) August precipitation sum (1920-2012), (b) August average temperature (1920-2012), and (c) MXD chronologies (1920-1978) with August NAJ positions for 2 degree longitudinal windows from 60 to 0W. Dashed horizontal lines indicate p<0.001 significance levels. Light grey zone indicates the latitudinal window selected for reconstruction (30-10W).

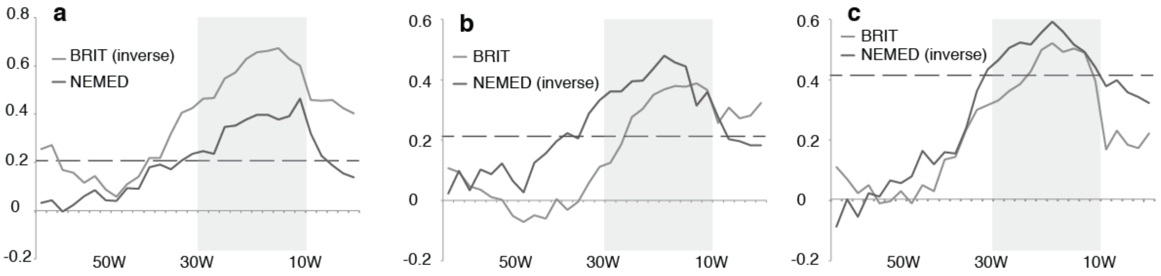

**Supplementary Figure 5:** Correlation coefficients of (a) NEMED and BRIT MXD chronologies with monthly (January to December) regional NEMED and BRIT temperature values (1901-1978) and (b) BRIT-NEMED composite with monthly (July, August, September, and August/September average) NAJ positions (1920-1978) for 2 degree longitudinal windows from 60 to 0W. Dashed horizontal lines indicate $p<0.001$ significance levels. Light grey zone in (b) indicates the latitudinal window selected for reconstruction (30-10W).

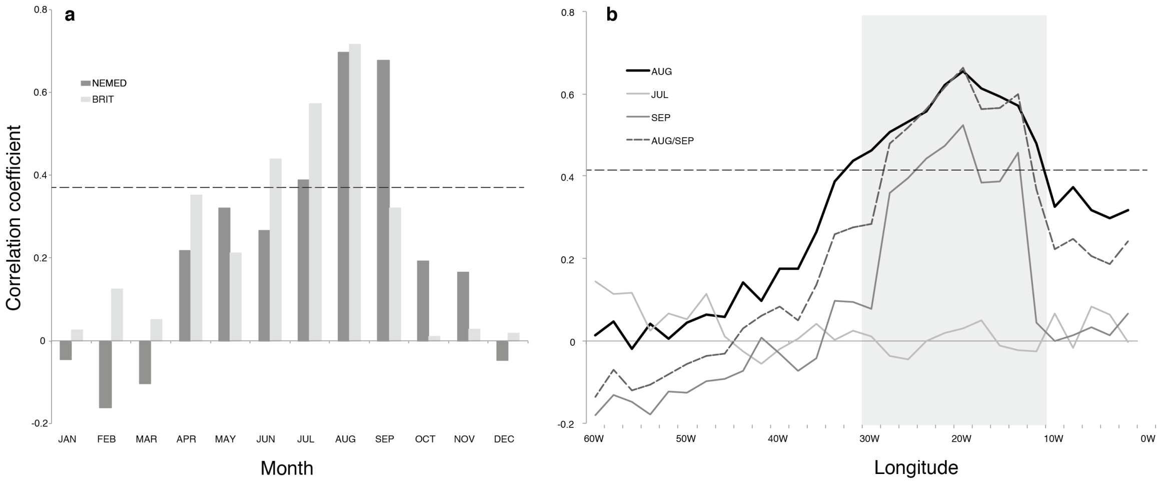

**Supplementary Figure 6**: (a) 20C Reanalysis August NAJ versus RPCA-based indices of summer NAO and (inversed) EA[3] (1950-2012). (b) annual and decadally smoothed (20 yr smoothing spline) reconstructions of August NAJ and of summer NAO[4].

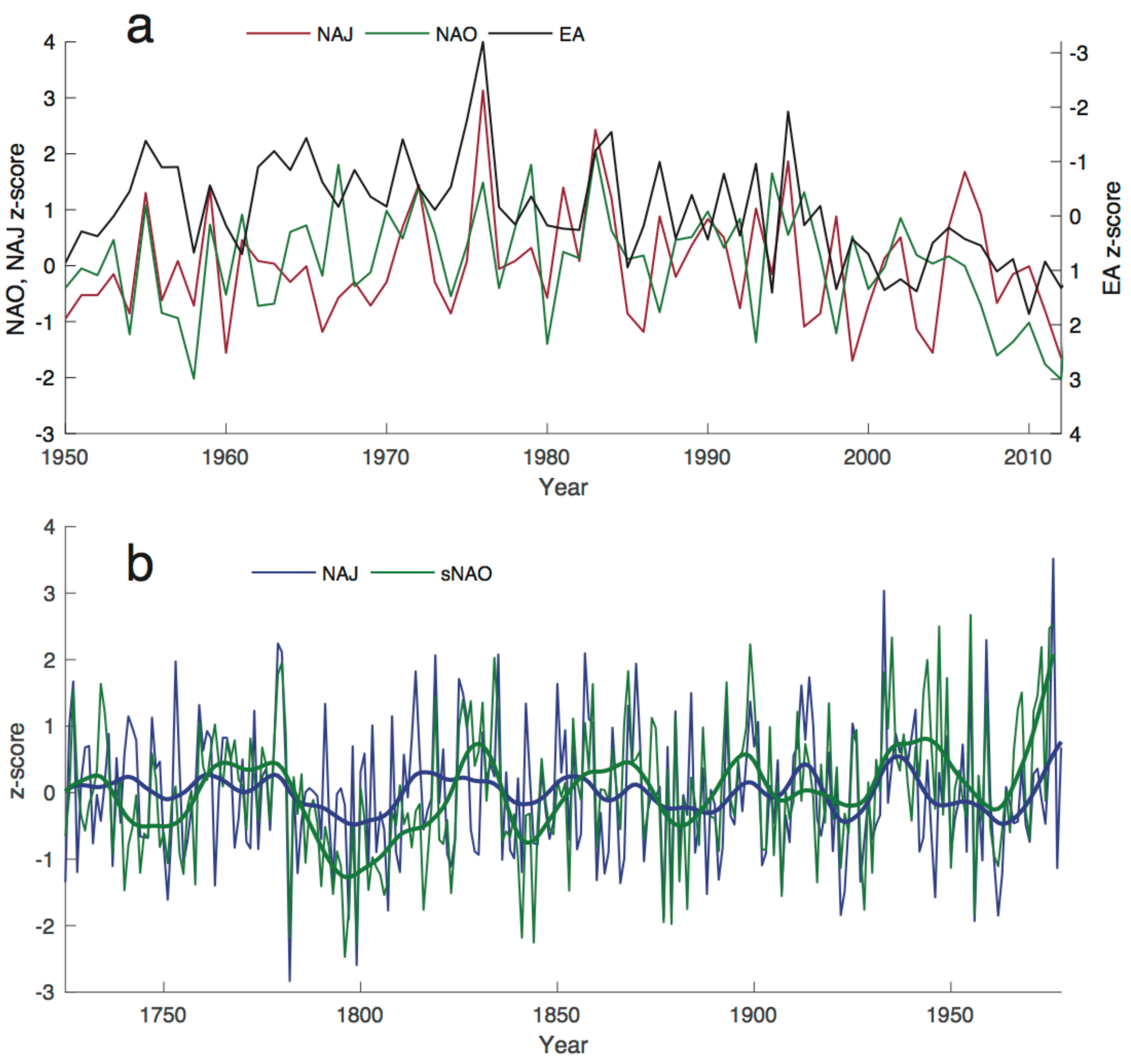

**Supplementary Figure 7**: Spectral analysis of the 20C Reanalysis (a; 1920-1978) and the reconstructed (b; 1725-1978) August NAJ time series. The 95% confidence interval for peaks in the power spectrum in a and c is indicated by the grey line. (c) Wavelet (Morlet 6) analysis of the August NAJ reconstruction (1725-1978). Bold black line indicates the cone of influence from edge effects. Statistically significant ($p<0.1$) peaks in the power spectrum in (c) are represented by colored shading.

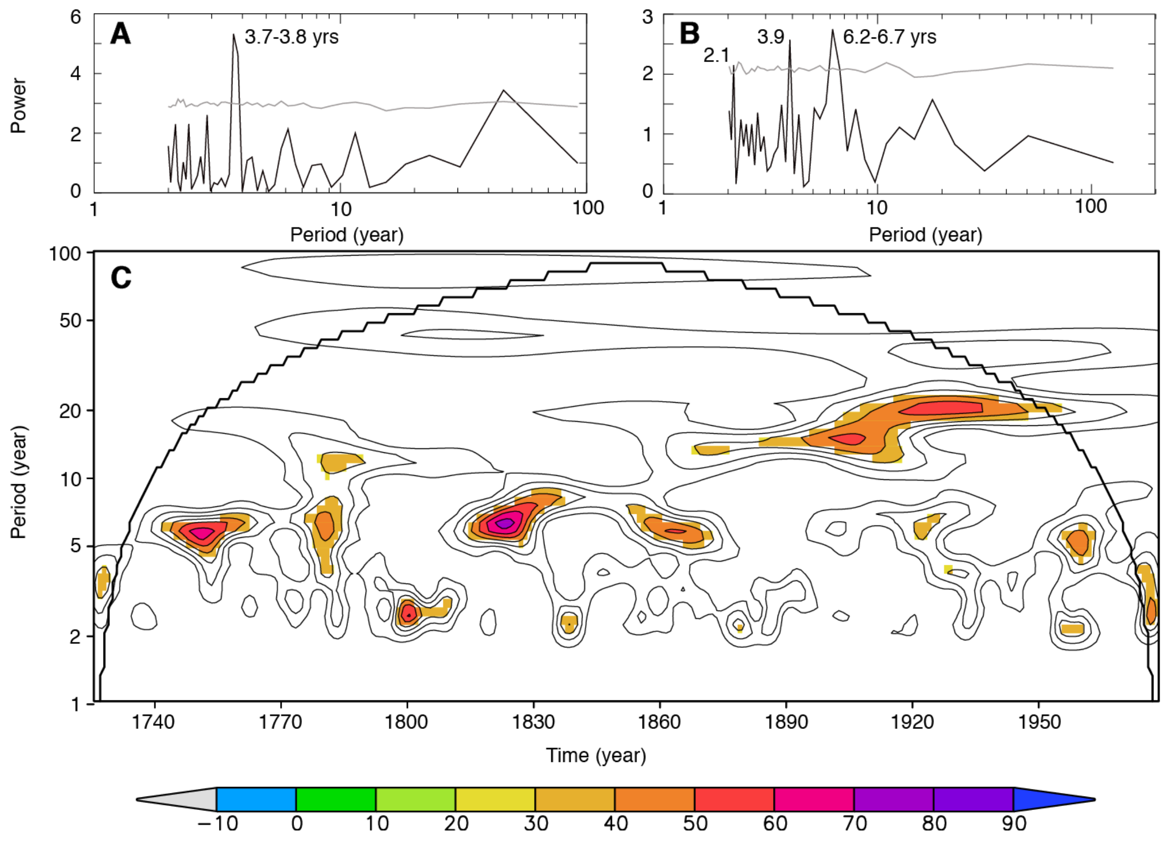

**Supplementary Figure 8**: Running 31-year window coefficients of variance (a), number of extreme years (b), and persistence of extremes (c) are plotted on the central year of the window for reconstructed and 20C Reanalysis August NAJ time series. NAJ reconstruction was scaled to match and mean of the 20CR-based August NAJ data over the period 1920-1978 (rather than 1948-1978 in Fig. 3). Extreme anomalies are defined as years when NAJ>|1stdev|, with standard deviation calculated based on a merged time series of reconstructed (1725-1919) and 20C Reanalysis (1920-1978) NAJ values. Horizontal dashed lines represent the highest 31-year values over the reconstruction period (1725-1978).

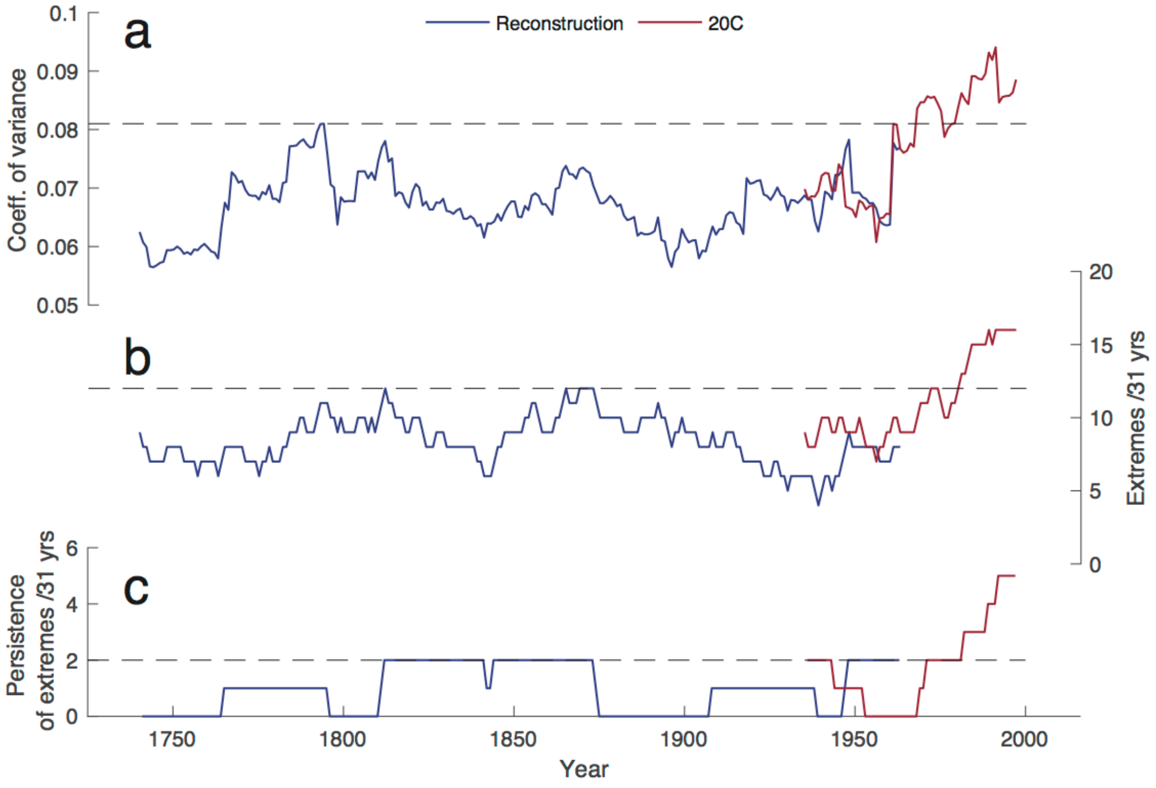

**Supplementary Figure 9**: Distribution of 20C Reanalysis derived versus reconstructed August NAJ positions (1920-1978 CE)

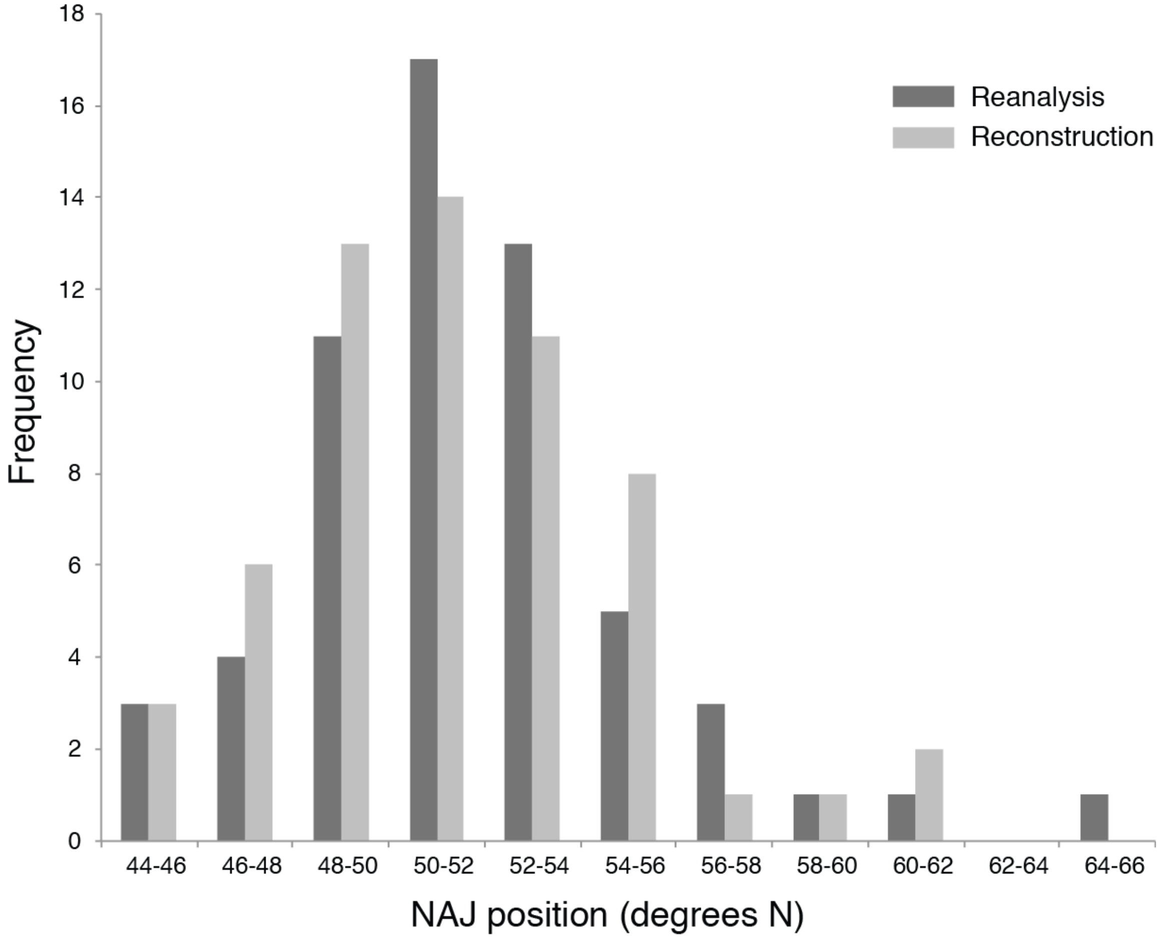

**Supplementary Figure 10**: Superposed Epoch Analyses (SEA) of SE Europe fire event series with NW Europe temperature (a) and precipitation (b). The fire event series is a tree-ring based fire record[5] from Mt. Taygetos, Greece (n=12; 1823-1940). Climate time series are Central England summer (June-August) temperature (a; 1725-2014) and England-Wales summer precipitation[6] (b; 1766-2014). Black symbols indicate statistical significance ($p<0.05$). The analysis window includes up to 5 years before and after each event year.

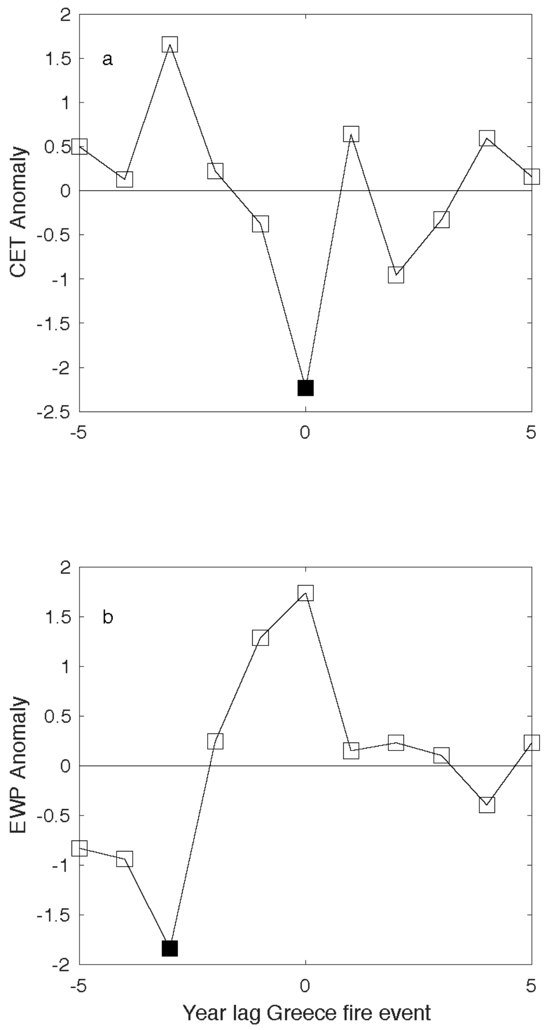

**Supplementary Table 1**: Site description for tree-ring sites in the British Isles (BRIT) and the northeastern Mediterranean (NEMED)

| Site | Latitude N | Longitude W | Elevation (masl) | Species | Chronology length (CE) | Number of series in compilation chronology |
|---|---|---|---|---|---|---|
| **BRIT** | | | | | | |
| Glen Affric[1] | 57.28 | 4.92 | 300 | PISY | 1735-1976 | 23 |
| Coulin[1] | 57.53 | 5.21 | 250 | PISY | 1671-1978 | 19 |
| Plockton[1] | 57.33 | 5.67 | 100 | PISY | 1879-1976 | 17 |
| Mallaig[1] | 56.97 | 5.75 | 20 | PISY | 1903-1976 | 15 |
| Inverey[1] | 57 | 3.58 | 500 | PISY | 1706-1976 | 24 |
| Shieldaig[1] | 57.5 | 5.62 | 12 | PISY | 1847-1978 | 19 |
| Ballochbuite[1] | 56.95 | 5.32 | 381 | PISY | 1712-1978 | 22 |
| Loch Maree[1] | 57.52 | 5.33 | 100 | PISY | 1756-1978 | 15 |
| Franchise Wood[1] | 50.95 | 1.68 | 100 | PISY | 1843-1976 | 15 |
| Glen Derry[1] | 57.02 | 3.57 | 457 | PISY | 1773-1978 | 24 |
| Drimmie[1] | 56.63 | 3.35 | 200 | PISY | 1828-1976 | 20 |
| **NEMED** | | | | | | |
| Cortina[1] d'Ampezzo S | 46.53 | 12.07 | 1900 | PCAB | 1660-1981 | 4 |
| Jahorina[1] | 43.75 | 18.63 | 1700 | PCAB | 1736-1981 | 24 |
| Cortina[1] d'Ampezzo | 46.53 | 12.07 | 1820 | PCAB | 1737-1980 | 5 |
| Vrsic[1] | 46.47 | 13.75 | 1600 | PCAB | 1757-1981 | 12 |

| | | | | | | |
|---|---|---|---|---|---|---|
| Novaci[1] | 45.3 | 23.67 | 1650 | PCAB | 1804-1981 | 29 |
| Vlasic[1] | 44.3 | 17.58 | 1600 | PCAB | 1823-1981 | 24 |
| Roncan[1] | 47.45 | 25.47 | 1480 | PCAB | 1836-1981 | 21 |
| Blumone[1] | 45.88 | 10.42 | 1650 | PCAB | 1840-1980 | 9 |
| Rajinac[1] | 44.83 | 15 | 1550 | PCAB | 1868-1981 | 22 |
| Capra[1] | 45.55 | 24.67 | 1600 | PCAB | 1884-1981 | 20 |
| Sipenski[1] | 42.73 | 25.33 | 1300 | PCAB | 1922-1981 | 9 |
| Bosque di Martense[1] | 42.68 | 13.43 | 1700 | ABAL | 1654-1980 | 24 |
| Gambarie[1] | 38.17 | 15.92 | 1850 | ABAL | 1790-1980 | 20 |
| Mt. Pollino[1] | 39.9 | 16.2 | 1720 | ABAL | 1800-1980 | 7 |
| Falterona[1] | 43.87 | 11.67 | 1450 | ABAL | 1827-1980 | 20 |
| Abetone[1] | 44.12 | 10.7 | 1400 | ABAL | 1846-1980 | 22 |
| Sierra de Crispo[1] | 39.9 | 16.23 | 2000 | PILE | 1441-1980 | 21 |
| Olympos[1] | 40.08 | 22.42 | 2250 | PILE | 1583-1981 | 29 |
| Katara Pass[1] | 39.8 | 21.22 | 1750 | PILE | 1673-1981 | 18 |
| Vihren[2] | 41.7 | 23.5 | 2300 | PILE | 1721-2008 | 36 |
| Cakor Pass[1] | 42.63 | 19.93 | 1750 | PCOM | 1856-1981 | 25 |

[1] Data contributed by F.H. Schweingruber; [2] Data from the International Tree-Ring Data Bank[7]

PISY= *Pinus sylvestris;* PCAB= *Picea abies;* ABAL= *Abies alba*; PILE= *Pinus leucodermis*; PCOM=*Picea omorika*

**Supplementary Table 2**: Calibration and verification statistics for the PCA1 chronology versus August NAJ position

| Period | Calibration | Verification | | |
|---|---|---|---|---|
| | $R^2$ | $R^2$ | RE | CE |
| **1920-1949** | 0.27 | 0.27 | 0.03 | 0.03 |
| **1950-1978** | 0.52 | 0.52 | 0.45 | 0.45 |
| **1920-1978** | 0.4 | | | |

**Supplementary References**


1 Mitchell, T. D. & Jones, P. D. An improved method of constructing a database of monthly climate observations and associated high-resolution grids. *I.J. Climatol.* **25**, 693-712 (2005).

2 Compo, G. P. *et al.* The twentieth century reanalysis project. *Q. J. R. Meteorol. Soc.* **137**, 1-28 (2011).

3 Barnston, A. G. & Livezey, R. E. Classification, seasonality and persistence of low-frequency atmospheric circulation patterns. *Mon. Weather Rev.* **115**, 1083-1126 (1987).

4 Folland, C. K. *et al.* The summer North Atlantic Oscillation: past, present, and future. *J. Clim.* **22**, 1082-1103 (2009).

5 Christopoulou, A., Fule, P. Z., Andriopoulos, P., Sarris, D. & Arianoutsou, M. Dendrochronology-based fire history of Pinus nigra forests in Mount Taygetos, Southern Greece. *For. Ecol. Manag.* **293**, 132-139 (2013).

6 Alexander, L. & Jones, P. Updated precipitation series for the UK and discussion of recent extremes. *Atmos. Sci. Lett.* **1**, 142-150 (2000).

7 Grissino-Mayer, H. D. & Fritts, H. C. The International Tree-Ring Data Bank: An enhanced global database serving the global scientific community. *Holocene* **7**, 235-238 (1997).
